# Association between height and hypertension among US adults: analyses of National Health and Nutrition Examination Survey 2007–18

**DOI:** 10.1186/s40885-021-00164-4

**Published:** 2021-02-26

**Authors:** Rajat Das Gupta, Maxwell Akonde, Ibrahim Hossain Sajal, Gulam Muhammed Al Kibria

**Affiliations:** 1 Department of Epidemiology and Biostatistics, Arnold School of Public Health, University of South Carolina, Columbia, SC USA; 2 BRAC James P Grant School of Public Health, BRAC University, Mohakhali, Dhaka, Bangladesh; 3 Department of Mathematical Sciences, School of Natural Sciences & Mathematics, University of Texas at Dallas, Dallas, TX USA; 4 Department of Epidemiology and Public Health, University of Maryland School of Medicine, Baltimore, MD USA

**Keywords:** Hypertension, Body height

## Abstract

**Background:**

Previous studies that investigated association of height with prevalence and control of hypertension found mixed results. This cross-sectional study explored these associations among US adults (≥20 years).

**Methods:**

The National Health and Nutrition Examination Survey (NHANES) 2007–18 data was analyzed. Height was measured in meters and was converted into centimeters (cm) and was further divided into quartiles: Q1 (135.3–159.2 cm), Q2 (159.3–166.2 cm), Q3 (166.3–173.6 cm), Q4 (173.7–204.5 cm). Hypertension definition of the ‘2017 American College of Cardiology/American Heart Association Guideline’ was used. Logistic regression analyses were conducted to find out the association between the dependent variable and the covariates. Linear regression analyses were conducted to find out the association of height with systolic blood pressure (SBP), diastolic blood pressure (DBP), pulse pressure (PP), and the covariates among the individuals who were not taking any antihypertensive drugs. Crude odds ratio, adjusted odds ratio (AOR), and adjusted beta-coefficient (for linear regression) with 95% confidence interval (CI) were reported. The following covariates were included: age, gender, race/ethnicity, family income, education level, cholesterol level, high-density lipoprotein level, chronic kidney disease status, diabetes status, smoker, aerobic leisure-time physical activity, and survey period. Sample weight of NHANES was adjusted.

**Results:**

Among the 21,935 participants (47.1% males), the prevalence of hypertension was 46.1%. Among 6154 participants taking medication (43.0% males), 57.2% had uncontrolled hypertension. In the final logistic regression analyses, participants in Q2 height quartile had 20% lower odds of being hypertensive compared to those in Q4 height quartile (AOR: 0.8; 95% CI: 0.7,1.0). Other height categories did not reveal any significant association. Compared to Q4 height category, Q1 (AOR: 1.7; 95% CI: 1.2,2.3), Q2 (AOR: 1.4; 95% CI: 1.1,1.8), and Q3 (AOR: 1.3; 95% CI: 1.1,1.6) height categories had higher odds of uncontrolled hypertension. PP was inversely associated and DBP was positively associated with height.

**Conclusions:**

Although height was not associated with prevalence of hypertension, it had inverse association with uncontrolled hypertension. It was also significantly associated with DBP and PP among the individuals with untreated hypertension.

**Supplementary Information:**

The online version contains supplementary material available at 10.1186/s40885-021-00164-4.

## Introduction

Hypertension is the largest risk factor contributing to global burden of mortality. According to the Global Burden of Disease Study 2017, around 10.4 million deaths occur every year due to hypertension [1]. Approximately 20.9% of the global annual Disability-Adjusted Life Years are lost due to hypertension [2]. Uncontrolled hypertension is a risk factor of several non-communicable diseases, including cardiovascular disease (CVD), cerebrovascular disease, chronic kidney disease (CKD), and hypertensive retinopathy [3]. Similar to other countries in the world, hypertension is a major public health problem in the US. According to the latest estimate from National Health and Nutrition Examination Survey (NHANES) 2017–18 data, more than 45% US adults aged ≥18 years could be hypertensive [4]. Less than one-fourth of the adults may have controlled blood pressure level [5].

There are several modifiable and non-modifiable risk factors of hypertension, including age, gender, and race/ethnicity [3]. Although this is not often considered, the association of height with prevalence and control of hypertension has been explored by several previous studies. Overall, these studies revealed mixed results. While several studies from China [6] and Nepal [7] revealed positive association, a study from Nigeria did not find any association among the adult population [8]. On the other hand, one study from Bangladesh revealed a positive association [9], while another study revealed no significant association [10]. In the US, Bourgeois et al. (2017) found that adult height is negatively associated with systolic blood pressure (SBP), pulse pressure (PP), and positively associated with diastolic blood pressure (DBP) [11]. However, they did not examine the association between hypertension in general with adult height. Moreover, they did not investigate the association between height and hypertension control. Given the inconsistency in the evidence regarding the association between adult height and hypertension in the context of US, this study aims to fulfill these gaps in the literature and investigated the association of height with prevalence and control of hypertension among US adults.

## Methods

### Data source

This cross-sectional study analyzed the 2007–2018 NHANES data. NHANES is a nationally representative biennial survey in the US implemented by National Center for Health Statistics (NCHS) under the Centers for Disease Control and Prevention. The NHANES utilized a multi-staged cluster sampling design. The details of NHANES including survey design, methodologies, sampling process, and variables included in the datasets were published previously [12, 13]. The datasets were downloaded and then merged using the unique identification numbers.

### Outcome variable

The eligible population for this study was men and women aged ≥ 20 years with ≥ 3 blood pressure measurements. Participants who don’t have at least 3 measurements (i.e., 1 or 2 measurements), we obtained BP levels from the available measurements. Blood pressure status was the outcome of interest in this study. Blood pressure was measured by trained research physicians using factory-calibrated Baumanometer® (W.A.Baum Co., Copiague, NY, USA) mercury true gravity wall model sphygmomanometers after the participants remained in seating position for at least 5 min. The appropriate cuff size was used [14].

Prevalence and control of hypertension was defined as per 2017 American College of Cardiology/American Heart Association (ACC/AHA) guideline. An individual was considered as hypertensive if any of the following three conditions were met: SBP ≥ 130 mm Hg, DBP ≥ 80 mm Hg or self-report of taking antihypertensive drugs [15]. Among individuals who reported that they were taking antihypertensive drugs, uncontrolled hypertension was defined as SBP ≥ 130 mm Hg and/or DBP ≥ 80 mm Hg [15].

### Study variables

Height/stature was the main explanatory variable of interest. It was measured in meters (m) standing position with a wall-mounted digital stadiometer [16]. During analyses, it was converted into centimeters (cm) and was further divided into quartiles: Q1 (135.3–159.2 cm), Q2 (159.3–166.2 cm), Q3 (166.3–173.6 cm), Q4 (173.7–204.5 cm) [6].

Based on literature review, the following covariates were considered: age, gender, race/ethnicity, family income, education level, cholesterol level, High-density lipoprotein (HDL) level, CKD status, diabetes status, smoker, leisure-time physical activity (LTPA), and survey period.

Age (in years), gender, race/ethnicity, family income, and education level were reported by the participants. Age was categorized into 20–39 years, 40–59 years, and ≥ 60 years. Gender was dichotomized into male and female. Race/ethnicity was categorized into non-Hispanic whites, non-Hispanic blacks, Mexican-Americans, and others. Family income was divided into three equal-sized strata: low, middle, and high. Education level was categorized into below high school, high school, and college graduate or above.

Cholesterol level was categorized into ‘no high cholesterol’ [< 200 mg/ deciliter (mg/dl)], ‘borderline elevated’ (200–239 mg/dl), ‘high cholesterol’ (≥240 mg/dl). Self-reported intake of cholesterol-lowering drugs were also included in the ‘high cholesterol’ group. HDL levels were categorized as low (< 40 mg/dl for men and < 50 mg/dl for women) and normal. CKD was defined as having an albumin-creatinine ratio ≥ 30 mg/gram (mg/g) or a glomerular filtration rate (GFR) < 60 ml/minute (ml/min) per 1.73 m^2^ (m^2^) [17]. Diabetes mellitus was defined as fasting plasma glucose ≥126 mg/dl or self-report of taking antidiabetic drugs.

Smoking status was dichotomized into yes and no. Participants were asked about the usual amount of time spent to perform moderate and vigorous aerobic recreational physical activity (PA) in a week. LTPA was calculated by summing the minutes spent to perform vigorous PA multiplied by two with the minutes spent to perform moderate PA. Aerobic LTPA was categorized into no (0 min/week), some (> 0 to < 150 min/week), and high (≥150 min/week) [5]. To increase sample size, the survey years were merged and categorized into 2007–2010, 2011–2014, and 2015–2018. Supplemental Table 1 describes study variables.

### Statistical analysis

Descriptive analyses were carried out and the findings were presented in unweighted frequencies and weighted percentages. Then, bivariate analyses were carried out to observe the distribution of the covariates according to hypertension status and according to height quartiles. Also, the uncontrolled hypertension status among those who were taking blood pressure lowering drugs were presented, according to the distribution of the selected covariates. Additionally, the distribution of covariates across the height quartiles among those who were taking blood pressure lowering drugs was presented. In order to find out the association of height with hypertension prevalence and control, unadjusted and multivariable logistic regression analyses were conducted. The variables which yielded a *p* < 0.05 (which was considered enough to control residual confounding in the multivariable model) in the crude analysis were included in the multivariable logistic regression analyses [18]. Both crude odds ratios (CORs) and adjusted odds ratios (AORs) were reported with 95% confidence intervals (CIs). With separate logistic regression models, we also examined the interaction between age and height. Lastly, we ran three separate multiple linear regression model considering SBP, DBP, and PP as outcomes and height as the main predictor among the individuals with who reported that they were not taking any antihypertensive drugs. As the proportion of missing data was low (< 10%), we used complete case analysis approach to handle missing data. During the analyses, the mobile examination center (MEC) weights of NHANES was used. All the analyses were done using Stata 14.0 (College Station, Texas, USA).

### Ethical approval

The National Center for Health Statistics approved the protocol for NHANES. Informed consent was taken from the participants before data collection. This study utilized NHANES data which are publicly available and de-identified, hence was deemed exempted from review by the institutions’ Institutional Review Board (IRB).

## Results

The background characteristics of the participants according to hypertension status is shown in Table 1. In the final analyses 21,935 participants were included. Among them 47.1% (*n* = 10,510) were males. The overall prevalence of hypertension was (49.1%). The proportion of hypertensive patients increased with age. Approximately three-fifths of the participants were non-Hispanic whites (62.8%), with almost equal proportion of non-Hispanic blacks (12.3%), Mexican-Americans (10.4%), and others races/ethnicities (14.5%). The proportion of respondents who were educated up to college or above (with hypertension: 23.4%, without hypertension: 16.9%), with normal HDL (with hypertension: 69.0%, without hypertension: 64.4%), and high LTPA (with hypertension: 39.3%, without hypertension: 26.5%) were higher among hypertensive people than people without hypertension. Comparison of respondents according to height is shown in Supplementary Table 2.

**Table 1:** Comparison of respondents according to hypertension status, NHANES 2007–18

Variables	Overall, % (n)	By Hypertension		
		No Hypertension, % (n)	Hypertension, % (n)	***p***-values
**Age (in year)**				
20–39	39.9 (7697)	18.5 (1659)	58.3 (6038)	< 0.001
40–59	34.5 (6959)	38.6 (3674)	31.0 (3285)	
60+	25.6 (7279)	42.9 (5830)	10.7 (1449)	
**Gender**				
Male	47.1 (10,499)	50.1 (5671)	44.6 (4828)	< 0.001
Female	52.9 (11,436)	49.9 (5492)	55.4 (5944)	
**Race/Ethnicity**				
Non-Hispanic White	62.8 (8798)	65.4 (4614)	60.6 (4184)	< 0.001
Non-Hispanic Black	12.3 (4714)	14.5 (2850)	10.5 (1864)	
Mexican-American	10.4 (3641)	7.8 (1560)	12.5 (2081)	
Other races/ethnicities	14.5 (4782)	12.3 (2139)	16.4 (2643)	
**Family income to poverty ratio**				
Low	25.6 (7533)	23.7 (3672)	27.2 (3861)	< 0.001
Middle	33.0 (7615)	34.2 (4047)	32.0 (3568)	
High	41.4 (6787)	42.0 (3444)	40.8 (3343)	
**Education level**				
Below High School	19.4 (6057)	21.3 (3399)	17.8 (2658)	< 0.001
High School	60.2 (12,241)	61.7 (6203)	58.9 (6038)	
College Graduate or Above	20.4 (3637)	16.9 (1561)	23.4 (2076)	
**Total cholesterol level**				
No high cholesterol	44.0 (9301)	30.6 (3388)	55.5 (5913)	< 0.001
Borderline elevated	17.8 (3823)	16.6 (1770)	18.8 (2053)	
High cholesterol	38.2 (8811)	52.8 (6005)	25.7 (2806)	
**High-density lipoprotein (in mg/dl)**				
Normal	66.9 (14,551)	64.4 (7257)	69.0 (7294)	< 0.001
Low	33.1 (7384)	35.6 (3906)	31.0 (3478)	
**Chronic kidney disease**				
No	84.7 (17,809)	75.2 (7930)	92.8 (9879)	< 0.001
Yes	15.3 (4126)	24.8 (3233)	7.2 (893)	
**Diabetes mellitus status**				
No	87.1 (18,260)	78.5 (8304)	94.4 (9956)	< 0.001
Yes	12.9 (3675)	21.5 (2859)	5.6 (816)	
**Smoker**				
No	76.9 (16,945)	79.1 (8857)	75.0 (8088)	< 0.001
Yes	23.1 (4990)	20.9 (2306)	25.0 (2684)	
**Leisure time physical activity (in minutes)**				
No/Low	50.9 (12,249)	58.3 (6874)	44.6 (5375)	0.20
Some (< 150 min)	15.7 (3134)	15.2 (1582)	16.0 (1552)	
High (≥150 min)	33.4 (6552)	26.5 (2707)	39.3 (3845)	
**Survey year**				
2007–10	31.9 (7791)	31.3 (3911)	32.4 (3880)	0.20
2011–14	34.3 (7236)	33.9 (3547)	34.6 (3689)	
2015–18	33.8 (6908)	34.8 (3705)	32.9 (3203)	
**Height categories**				
Q1 (135.3–159.2)	20.6 (5476)	22.7 (2941)	18.8 (2535)	< 0.001
Q2 (159.3–166.2)	24.8 (5460)	22.9 (2641)	26.4 (2819)	
Q3 (166.3–173.6)	25.0 (5474)	24.3 (2764)	25.7 (2710)	
Q4 173.7–204.5)	29.5 (5525)	30.0 (2817)	29.1 (2708)	

Numbers are presented as weighted column percentages and unweighted numbers, *p*-values were obtained with chi-square tests

*NHANES* National Health and Nutrition Examination Survey

Table 2 compares respondents according to uncontrolled hypertension status among those who were taking blood pressure lowering drugs. In total 6154 participants were taking blood pressure lowering drugs. About 57.2% people had uncontrolled hypertension. Among people with uncontrolled hypertension, the proportions of respondents with ≥60 years of age (61.4%), non-Hispanic black race/ethnicity (18.0%), low family income (24.5%), having education level below high school (24.0%), and with CKD (37.0%), were higher compared to people with controlled hypertension. Regarding the height category, greater proportion from Q1 (controlled hypertension: 25.1%, uncontrolled hypertension: 29.5%) and lesser proportion of Q4 group (controlled hypertension: 28.0%, uncontrolled hypertension: 23.1%) had uncontrolled hypertension. Comparison of respondents according to height among those who were taking blood pressure lowering drugs is shown in Supplementary Table 3.

**Table 2:** Comparison of respondents according to uncontrolled hypertension status among those who were taking blood pressure lowering drugs, NHANES 2007–18

Variables	Overall, % (n)	Uncontrolled Hypertension		
		Yes, % (n)	No, % (n)	***p***-values
**Age (in year)**				
20–39	6.1 (292)	5.9 (168)	6.3 (124)	0.009
40–59	35.1 (1762)	32.8 (1009)	38.2 (753)	
60+	58.8 (4100)	61.4 (2595)	55.5 (1505)	
**Gender**				
Male	43.0 (2768)	43.2 (1718)	42.9 (1050)	0.88
Female	57.0 (3386)	56.8 (2054)	57.1 (1332)	
**Race/Ethnicity**				
Non-Hispanic White	67.7 (2638)	64.4 (1497)	72.2 (1141)	< 0.001
Non-Hispanic Black	15.5 (1705)	18.0 (1134)	12.2 (571)	
Mexican-American	5.7 (732)	6.0 (451)	5.2 (281)	
Other races/ethnicities	11.1 (1079)	11.6 (690)	10.4 (389)	
**Family income to poverty ratio**				
Low	23.1 (2009)	24.5 (1260)	21.2 (749)	< 0.001
Middle	35.1 (2236)	36.4 (1385)	33.3 (851)	
High	41.8 (1909)	39.1 (1127)	45.5 (782)	
**Education level**				
Below High School	22.6 (1946)	24.0 (1249)	20.8 (697)	< 0.001
High School	61.8 (3403)	62.2 (2057)	61.2 (1346)	
College Graduate or Above	15.6 (805)	13.8 (466)	18.0 (339)	
**Cholesterol level**				
No high cholesterol	22.6 (1422)	23.6 (891)	21.2 (531)	0.002
Borderline elevated	10.7 (644)	12.1 (427)	8.7 (217)	
High cholesterol	66.8 (4088)	64.3 (2454)	70.1 (1634)	
**High-density lipoprotein**				
Normal	62.1 (3884)	63.4 (2445)	60.3 (1439)	0.12
Low	37.9 (2270)	36.6 (1327)	39.7 (943)	
**Chronic kidney disease**				
No	66.7 (3860)	63.0 (2248)	71.6 (1612)	< 0.001
Yes	33.3 (2294)	37.0 (1524)	28.4 (770)	
**Diabetes mellitus status**				
No	68.2 (3944)	68.2 (2414)	68.0 (1530)	0.92
Yes	31.8 (2210)	31.8 (1358)	32.0 (852)	
**Smoker**				
No	82.3 (5081)	83.8 (3150)	80.2 (1931)	0.007
Yes	17.7 (1073)	16.2 (622)	19.8 (451)	
**Leisure time physical activity (in minutes)**				
No	63.3 (4034)	63.6 (2475)	62.8 (1559)	0.70
Some (< 150 min)	14.7 (872)	14.2 (530)	15.3 (342)	
High (≥150 min)	22.1 (1248)	22.2 (767)	21.9 (481)	
**Survey year**				
2007–10	31.4 (2179)	31.7 (1309)	30.9 (870)	0.033
2011–14	34.6 (1989)	32.7 (1188)	37.2 (801)	
2015–18	34.0 (1986)	35.6 (1275)	31.9 (711)	
**Height categories**				
Q1 (135.3–159.2)	27.6 (1869)	29.5 (1200)	25.1 (669)	0.009
Q2 (159.3–166.2)	24.3 (1506)	24.2 (921)	24.4 (585)	
Q3 (166.3–173.6)	22.9 (1444)	23.2 (879)	22.5 (565)	
Q4 173.7–204.5)	25.2 (1335)	23.1 (772)	28.0 (563)	

Numbers are presented as weighted column percentages and unweighted numbers, *p*-values were obtained with chi-square tests

*NHANES* National Health and Nutrition Examination Survey

The association of height with presence of hypertension is presented in Table 3. In adjusted analyses, participants in Q2 height quartile had 20% less odds of being hypertensive compared to those in Q4 height quartile (AOR: 0.8; 95% CI: 0.7,1.0). Other height categories did not reveal any significant association. Older age (40–59 years and ≥ 60 years), non-Hispanic black race/ethnicity, borderline elevated and high serum cholesterol levels, presence of CKD, and diabetes mellitus were also positively associated with hypertension. On the other hand, female gender, Mexican-American race/ethnicity, being college graduate or above, and having LTPA ≥150 min were inversely associated with hypertension.

**Table 3:** Association of height with prevalence of hypertension, NHANES 2007–18

Variables	COR (95% CI)	AOR (95% CI)
**Height Category (in cm)**		
Q1 (135.3–159.2)	1.2** (1.0, 1.3)	1.0 (0.8,1.2)
Q2 (159.3–166.2)	0.8*** (0.8, 0.9)	0.8* (0.7,1.0)
Q3 (166.3–173.6)	0.9 (0.8,1.0)	0.9 (0.8,1.0)
Q4 173.7–204.5)	Ref.	Ref.
**Age (in year)**		
20–39	Ref.	Ref.
40–59	3.9*** (3.6,4.3)	3.1*** (2.8,3.4)
≥60	12.7*** (11.3,14.2)	7.8*** (6.8,8.9)
**Gender**		
Male	Ref.	Ref.
Female	0.8*** (0.7,0.9)	0.7*** (0.6,0.8)
**Race/Ethnicity**		
Non-Hispanic White	Ref.	Ref.
Non-Hispanic Black	1.3*** (1.2,1.4)	1.8***(1.7,2.0)
Mexican-American	0.6*** 0.5,0.7)	0.7***(0.7,0.8)
Other races/ethnicities	0.7*** (0.6,0.8)	0.8**(0.8,1.0)
**Family income**		
Low	Ref.	Ref.
Middle	1.2*** (1.1,1.3)	1.1 (0.9,1.2)
High	1.2*** (1.1,1.3)	1.1 (1.0,1.2)
**Education level**		
Below High School	Ref.	Ref.
High School	0.9** (0.8,1.0)	1.0 (0.9,1.1)
College Graduate or Above	0.6*** (0.5,0.7)	0.7*** (0.6,0.9)
**Cholesterol level (in mg/dl)**		
No high cholesterol	Ref.	Ref.
Borderline elevated	1.6*** (1.4,1.8)	1.3***(1.2,1.5)
High cholesterol	3.7*** (3.4,4.0)	1.7***(1.6,1.9)
**High-density lipoprotein cholesterol (in mg/dl)**		
Normal	Ref.	Ref.
Low	1.2*** (1.1,1.3)	1.3*** (1.2,1.5)
**Chronic kidney disease**		
No	Ref.	Ref.
Yes	4.3*** (3.9,4.7)	2.2*** (1.9,2.5)
**Diabetes mellitus status**		
No	Ref.	Ref.
Yes	4.6*** (4.0,5.2)	1.8*** (1.6,2.1)
**Smoker**		
No	Ref.	Ref.
Yes	0.8*** (0.7,0.9)	0.9 (0.8,1.0)
**Leisure time physical activity (in minutes)**		
No	Ref.	Ref.
Some (< 150 min)	0.4*** (0.3,0.5)	0.9 (0.8,1.0)
High (≥150 min)	0.4*** (0.3,0.4)	0.8***(0.7,0.9)
**Survey Period**		
2007–10	Ref.	Ref.
2011–14	1.0 (0.9,1.1)	1.0 (0.9,1.1)
2015–18	1.1 (1.0,1.2)	1.1 (0.9,1.3)

Adjusted for all variables in the column, variables that were significant in bivariate analysis were included into the multivariable analysis

*AOR* Adjusted Odds Ratio, *COR* Crude Odds Ratio, *NHANES* National Health and Nutrition Examination Survey

*p < 0.05, **p < 0.01, ***p < 0.001

Table 4 shows the logistics regression analyses to find out the association of height with uncontrolled hypertension. Although age and gender were not significantly associated in the bivariate analyses, we have adjusted for them in the multivariable analysis based on findings from previous literature. In the final multivariable model, among those who were taking antihypertensive medications, the odds of having uncontrolled hypertension were 70%, 40% and 30% higher among Q1 (AOR: 1.7; 95% CI: 1.2,2.3), Q2 (AOR: 1.4; 95% CI: 1.1,1.8), and Q3 (AOR: 1.3; 95% CI: 1.1,1.6) height quartile respectively, compared to those who were in Q4 height quartile. Among other studied association, non-Hispanic black race/ethnicity, and CKD status were positively associated with uncontrolled hypertension. On the other hand, female gender, and smoking were inversely associated with uncontrolled hypertension. Interaction between height and age found that those who were 60+ years old and were in Q4 height quartile had 70% lower odds of having uncontrolled hypertension (AOR: 0.3; 95% CI: 0.1,0.6) compared to those who were 20–39 years old and were in Q1 height quartile (Supplementary Table 4).

**Table 4:** Association of height with uncontrolled hypertension among antihypertensive drug users, NHANES 2007–18

Variables	COR (95% CI)	AOR (95% CI)
**Height Category (in cm)**		
Q1 (135.3–159.2)	1.4** (1.1,1.8)	1.7*** (1.2,2.3)
Q2 (159.3–166.2)	1.2 (1.0,1.5)	1.4* (1.1,1.8)
Q3 (166.3–173.6)	1.3* (1.0,1.5)	1.3* (1.1,1.6])
Q4 (173.7–204.5)	Ref.	Ref.
**Age (in year)**		
20–39	Ref.	Ref.
40–59	0.9 (0.7,1.2)	0.9 (0.7,1.2)
≥60	1.2 (0.9,1.6)	1.0 (0.7143)
**Gender**		
Male	Ref.	Ref.
Female	1.0 (0.8,1.2)	0.7*** (0.6,0.9)
**Race/Ethnicity**		
Non-Hispanic White	Ref.	Ref.
Non-Hispanic Black	1.7*** (1.4,1.9)	1.7*** (1.4,2.0)
Mexican-American	1.3** (1.1,1.6)	1.1 (0.9,1.4)
Other races/ethnicities	1.3* (1.0,1.5)	1.2 (0.9,1.4)
**Family income**		
Low	Ref.	Ref.
Middle	0.9 (0.8,1.1)	1.0 (0.8,1.2)
High	0.7*** (0.6,0.9)	0.8 (0.7,1.0)
**Education level**		
Below High School	Ref.	Ref.
High School	0.9 (0.8,1.0)	1 (0.9,1.2)
College Graduate or Above	0.7*** (0.5,0.8)	0.8 (0.6,1.0)
**Cholesterol level (in mg/dl)**		
No high cholesterol (< 200)	Ref.	–
Borderline elevated (200–239)	1.3 (0.9,1.7)	–
High cholesterol (≥240)	0.8 (0.7,1.0)	–
**High-density lipoprotein cholesterol (in mg/dl)**		
Normal	Ref.	–
Low	0.9 (0.7,1.0)	–
**Chronic kidney disease**		
No	Ref.	Ref.
Yes	1.5*** (1.3,1.7)	1.3*** (1.1,1.6)
**Diabetes mellitus status**		
No	Ref.	–
Yes	1 (0.8,1.2)	–
**Smoker**		
No	Ref.	Ref.
Yes	0.8** (0.7,0.9)	0.8** (0.6,0.9)
**Leisure time physical activity (in minutes)**		
No	Ref.	
Some (< 150 min)	1 (0.6,1.7)	–
≥150 min	1.1 (0.8,1.6)	–
**Survey Period**		
2007–10	Ref.	Ref.
2011–14	0.9 (0.7,1.0)	0.9 (0.7, 1.0)
2015–18	1.1 (0.9,1.3)	1.1 (0.9, 1.3)

Adjusted for all variables in the column, variables significant in bivariate analyses were included in multivariable model

*AOR* Adjusted Odds Ratio, *COR* Crude Odds Ratio, *NHANES* National Health and Nutrition Examination Survey

*p < 0.05, **p < 0.01, ***p < 0.001

Supplementary Table 5 shows stratification of the association between height and hypertension according to age. For the 20–39 years age group, the odds of hypertension increased significantly with increasing height and for the 60+ years age group, the odds of the odds of hypertension decreased significantly with increasing height. For the 40–59 years old strata, there were no significant association.

Table 5 shows the adjusted beta coefficients (aβ) of SBP, DBP, and PP with height derived through adjusted linear regression analyses among the untreated individuals. In the final multiple regression model, SBP, and PP decreased, and DBP increased with increasing height, the association was significant for DBP, and PP only. The SBP was 0.7 mmHg, 0.87 mmHg, and 0.51 mmHg less among Q2 (aβ: − 0.70; 95% CI: − 1.59,0.19), Q3 (aβ: -0.87; 95% CI: − 1.87,0.13), and Q4 (aβ: -0.51; 95% CI: − 1.61,0.60) height quartile respectively, compared to those who were in Q1 height quartile. The DBP was 0.73 mmHg, 1.29 mmHg, and 1.92 mmHg higher among Q2 (aβ: 0.73; 95% CI: 0.14,1.31), Q3 (aβ: 1.29; 95% CI: 0.58,2.01), and Q4 (aβ: 1.92; 95% CI: 1.06,2.77) height quartile respectively, compared to those who were in Q1 height quartile. The PP was 1.43 mmHg, 2.16 mmHg, and 2.43 mmHg less among Q2 (aβ: -1.43; 95% CI: − 2.23,-0.63), Q3 (aβ: -2.16; 95% CI: − 3.08,-1.23), and Q4 (aβ: -2.43; 95% CI: − 3.54,-1.32) height quartile respectively, compared to those who were in Q1 height quartile.

**Table 5:** Adjusted Association of Systolic Blood Pressure, Diastolic Blood Pressure, and Pulse Pressure with Height derived through Linear Regression among the untreated individuals, NHANES 2007–18^#^

Variables^##^	SBP	DBP	PP
	aβ (95% CI)	aβ (95% CI)	aβ (95% CI)
**Height Category (in cm)**			
Q1 (135.3–159.2)	Ref	Ref	Ref
Q2 (159.3–166.2)	-0.7 (− 1.59,0.19)	0.73* (0.14,1.31)	-1.43*** (−2.23,-0.63)
Q3 (166.3–173.6)	−0.87 (− 1.87,0.13)	1.29*** (0.58,2.01)	− 2.16*** (−3.08,-1.23)
Q4 (173.7–204.5)	− 0.51 (− 1.61,0.60)	1.92*** (1.06,2.77)	− 2.43*** (− 3.54,-1.32)
**Age (in year)**			
20–39	Ref	Ref	Ref
40–59	5.42*** (4.73,6.12)	4.69*** (4.20,5.17)	0.74** (0.22,1.26)
≥60	14.24*** (13.23,15.24)	−0.59 (−1.37,0.18)	14.75*** (13.82,15.67)
**Gender**			
Male	Ref	Ref	Ref
Female	−5.96*** (−6.67,-5.26)	−2.11*** (−2.71,-1.52)	−3.88*** (−4.53,-3.23)
**Race/Ethnicity**			
Non-Hispanic White	Ref	Ref	Ref
Non-Hispanic Black	4.06*** (3.17,4.96)	0.77* (0.01,1.52)	3.31*** (2.24,4.37)
Mexican-American	−0.46 (− 1.35,0.43)	− 0.61 (− 1.49,0.28)	0.16 (− 0.69,1.01)
Other races/ethnicities	−0.73* (− 1.44,-0.01)	−0.1 (− 0.85,0.66)	−0.63(− 1.48,0.22)
**Family income**			
Low	Ref	Ref	Ref
Middle	0.19 (−0.51,0.89)	−0.04 (− 0.59,0.51)	0.23 (− 0.44,0.90)
High	0.16 (− 0.58,0.90)	0.09 (− 0.57,0.74)	0.09 (− 0.60,0.79)
**Education level**			
Below High School	Ref	Ref	Ref
High School	−0.4 (−1.12,0.32)	0.76** (0.22,1.30)	−1.13*** (− 1.72,-0.55)
College Graduate or Above	−2.67*** (−3.66,-1.67)	0.41 (− 0.32,1.15)	−3.06*** (− 3.88,-2.24)
**Cholesterol level (in mg/dl)**			
No high cholesterol (< 200)	Ref	Ref	Ref
Borderline elevated (200–239)	2.20*** (1.50,2.91)	2.47*** (1.98,2.96)	−0.26 (− 0.96,0.45)
High cholesterol (≥240)	1.77*** (0.95,2.59)	1.83*** (1.35,2.32)	−0.09(− 0.79,0.60)
**High-density lipoprotein (in mg/dl)**			
Normal	Ref	Ref	Ref
Low	1.19*** (0.58,1.80)	1.01*** (0.59,1.44)	0.18 (−0.35,0.71)
**Chronic kidney disease**			
No	Ref	Ref	Ref
Yes	5.94*** (4.63,7.24)	1.68*** (0.89,2.47)	4.24*** (3.28,5.20)
**Diabetes mellitus status**			
No	Ref	Ref	Ref
Yes	1.09* (0.00,2.18)	−0.98* (−1.78,-0.18)	2.07*** (1.06,3.07)
**Smoker**			
No	Ref	Ref	Ref
Yes	0.35 (−0.27,0.96)	−0.89** (−1.46,-0.31)	1.24*** (0.69,1.80)
**Leisure time physical activity (in minutes)**			
No	Ref	Ref	Ref
Some (< 150 min)	− 0.16 (− 0.91,0.58)	0.05 (− 0.60,0.71)	−0.21 (− 0.91,0.49)
≥150 min	−0.90** (− 1.45,-0.35)	−0.52* (− 1.04,-0.01)	−0.38 (− 0.94,0.19)
**Survey Period**			
2007–10	Ref	Ref	Ref
2011–14	0.46 (−0.39,1.32)	0.48 (−0.52,1.48)	0.01 (−1.02,1.05)
2015–18	1.96*** (1.13,2.79)	1.24* (0.13,2.35)	0.74 (−0.29,1.77)

*aβ* Adjusted Beta Co-efficient, *DBP* Diastolic Blood Pressure, *NHANES* National Health and Nutrition Examination Survey, *PP* Pulse Pressure, *SBP* Systolic Blood Pressure

#Individuals who were not taking any antihypertensive drugs were included in the analyses

##Adjusted for all variables in the column, variables significant in bivariate analyses were included in multivariable mode

*p < 0.05, **p < 0.01, ***p < 0.001

## Discussion

This current study attempts to find out the association of height with hypertension, uncontrolled hypertension, and BP levels as per among US adults. Using a nationally representative sample of 21,935 adults aged ≥20 years, the study found that there is an inverse association between height and uncontrolled hypertension among those who were taking antihypertensive medications. The association was significant across all the height quartiles compared to Q4 height category group. PP was inversely associated and DBP was positively associated with height; however, SBP did not have any significant association, among the untreated individuals with hypertension. There was also an interaction between older age and Q4 height category group among the individuals with uncontrolled hypertension. We did not observe any significant association between presence of hypertension and most height quartiles.

Previous studies have demonstrated the association between height and hypertension. The first association was explored in US young adults by Voors et al. (1982), which identified a positive association [19]. However, the result was difficult to interpret as the body size was adjusted during the analyses which was correlated with adult height, therefore, the body size/weight could be a mediator instead of confounder in the association between height and BP [11]. Later studies identified inverse association between height and SBP and PP and positive association between height and DBP [11]. London et al. after adjusting for BMI found larger SBP among the shorter participants compared to larger participants [20]. A prospective birth cohort study conducted among middle-aged men and women by Langenberg et al. found an inverse association of height with SBP and PP and a null association with DBP [21]. Several Asian studies also found this association. Das Gupta et al. (2019) found that for each 10 cm increase in height, the odds of hypertension decreases 10% in adult Nepalese population [7]. Another study from Bangladesh demonstrated that the participant’s whose height was in Q4 (≥1.673 m), had 18% less odds of being hypertensive compared to the participants from Q1 (≤1.51 m) [9]. A study from China carried on participants aged 37–94 years also found 20 and 17% less odds of hypertension respectively among male and female participants of Q4 height category (< 1.62 m for male and < 1.52 m for female) compared to Q1 height category (> 1.70 m for male and > 1.60 m for female) [6]. Studies from South American setting (Brazil) also revealed this association. Sichieri et al. (1999) included 2802 adults aged 20–65 years and found that that height was inversely associated with hypertension in females, but not in males [22]. Florêncio et al. (2004) found the same results among adults aged 18–60 years [23]. In our study, we found a significant inverse association for Q2 height group only. This association may be spurious and may be due to the differences in sample characteristics including racial/ethnic difference, sample size and definition of hypertension. Also, we found the inverse association between height and uncontrolled hypertension among the antihypertensive medication users, which was previously unexplored, to the best of our knowledge. Like Bourgeois et al. (2017), we found an inverse association with height and PP, and a positive association between height and DBP, however in the group with untreated hypertension [11]. In order to achieve blood pressure control, US health promotion programs should focus on individuals with short stature.

Several biological theories explain the inverse association between height and hypertension. Apart from genetic factors, anatomical factors also contribute to this. As height increases, the diameter of the coronary vessels also increases. This reduces the risk of atherosclerosis and hypertension [24]. Also, poorer lung function in the short stature people compared to long stature people acts as an important cause [25]. Although the association is unclear, the dynamic properties of the arterial tree could also contribute to this association. Short stature individuals have shorter atrial tree compared to long stature individual [26]. In short stature individuals, the earlier arrival of the reflected wave in the central aorta increases both central pressure and PP in the later part of the systole [27].

In addition, maternal and child nutrition, environmental factors, parental smoking habit, and socio-economic status are associated with adult height. These factors are also associated with subsequent development of hypertension [28, 29]. Due to cross-sectional nature of NHANES, this study did not explore the above-mentioned factors, which reiterates the necessity of future longitudinal studies in the context of the USA. However, we included family income, smoking, and LTPA as proxy indicators. This study also highlights the importance of child nutrition promotion programs. The process of stunting starts in the utero and most of the process completes before the age of 2 years. After that time, it is difficult to achieve catch-up growth [30]. Public health nutrition programs of US should focus on maternal nutrition and infant and young child feeding (IYCF).

Previous studies have shown the importance of controlling hypertension. Ettehad et al. (2016) in their systematic review and meta-analyses showed 20% reduction in the risk of major CVDs for every 10 mmHg reduction in SBP. The risk of coronary heart disease, stroke, and heart failure reduced 17, 27 and 28% respectively [31]. Bundy et al. (2017) in their meta-analyses of 42 randomized controlled trials found that when the SBP is 120–124 mmHg, the risk of CVD is the lowest [32]. The SBP/DBP levels of 130/80 mmHg was selected based on the trials’ inclusion and exclusion criteria as well as taking into account the careful measurement of the BP in the controlled environment of the trials compared to the clinical practice [33]. Individuals with short stature had higher risk of uncontrolled hypertension, they are more likely to suffer from these cardiovascular complications. Although this finding is already known, we have reconfirmed the significance of height in the context of controlling hypertension using the new 2017 ACC/AHA cutoff, especially after controlling for multiple factors including age, gender, race/ethnicity, income, and several comorbidities. Shorter individuals could be recommended to take more preventive and control measures including more physical exercise, balanced diet, and regular BP monitoring.

In the final multivariable model, the study reiterated the previously known risk factors of hypertension including older age [34], male gender [34], non-Hispanic black race/ethnicity [35], less educational attainment [35], high cholesterol [35], CKD [35], diabetes mellitus [35], and low aerobic LTPA [35]. US health promotion programs should focus on these factors for the prevention of hypertension. The poor control of hypertension among the non-Hispanic blacks is may be due to poor medication adherence and poor control of other risk factors (e.g., LTPA and body weight) for hypertension [5]. However, in this case we did not analyze the data on medication adherence. CKD is associated with hypertension and increases the odds of uncontrolled hypertension even in case of medication use [3]. High family income and being educated college graduate or above were inversely associated with uncontrolled hypertension. This may be due to increased awareness, behavioral differences, and access to health care delivery system, which increased medication adherence and ultimately blood pressure control [36].

This study has several notable strengths. Due to utilization of a nationally representative sample, the findings of this study are generalizable to the target population (i.e.: adults aged ≥20 years) of US. NHANES utilized validated questionnaire and calibrated instruments to measure the variables. However, the limitations of the study also warrant discussion. Due to cross-sectional nature of the survey, the temporal relationship between the explanatory variables and the outcome could not be established. NHANES measured the BP in a single point, while 2017 ACC/AHA guideline recommended measurement in a longitudinal setting [15]. NHANES (2007–2016) evaluated treatment status based on previous guidelines. As a result, the prevalence of unmet treatment goals and untreated individuals may be overestimated.

## Conclusion

This study found a significant association between height and uncontrolled hypertension. This finding is important for the people with short stature that the uncontrolled hypertension can increase their risk of several other diseases, including CVD, CKD, and stroke. Though the mechanism is unclear, these findings signify the management of hypertension among shorter individuals.

## Supplementary Information

**Additional file 1: Supplemental Table 1.** Description of study variables.

**Additional file 2: Supplementary Table 2.** Comparison of respondents according to height.

**Additional file 3: Supplementary Table 3.** Comparison of respondents according to height among those who were taking blood pressure lowering drugs, NHANES 2007-18.

**Additional file 4: Supplemental Table 4.** Interaction between height and age to investigate association of height with hypertension and untreated hypertension, NHANES 2007-18.

**Additional file 5: Supplemental Table 5.** Adjusted Odds Ratio of association of height with hypertension stratified by age, NHANES 2007-18.

## Data Availability

All the datasets of NHANES are openly available and can be accessed from the following link: https://wwwn.cdc.gov/nchs/nhanes/.

## Abbreviations

ACC/AHA: American College of Cardiology/American Heart Association
AOR: Adjusted Odds Ratio
BMI: Body Mass Index
CKD: Chronic Kidney Disease
COR: Crude Odds Ratio
CVD: Cardiovascular Disease
DBP: Diastolic Blood Pressure
HDL: High-Density Lipoprotein
LTPM: Leisure-Time Physical Activity
NCHS: National Center for Health Statistics
NHANES: National Health and Nutrition Examination Survey
SBP: Systolic Blood Pressure
