# Supplementary material for: Association between height and hypertension among US adults: analyses of National Health and Nutrition Examination Survey 2007–18
Source: Clin Hypertens. 2021 Feb 26;27:6. doi: 10.1186/s40885-021-00164-4 (PMC7908753; doi:10.1186/s40885-021-00164-4)
Supplement: Supplementary file 1 — Additional file 1: Supplemental Table 1. Description of study variables. [file 40885_2021_164_MOESM1_ESM.docx]

**Supplemental Table 1: Description of study variables**

| **Variable Name** | **Definition** |
| --- | --- |
| Age | Self-report of age. |
| Gender | Self-report of gender, male or female. |
| Race/Ethnicity | Self-report, grouped as non-Hispanic whites, non-Hispanic blacks, Mexican-American, and others. |
| Family income to poverty ratio | Based on the total family income and number of family members as per the federal poverty threshold. |
| Hypertension | Hypertension was defined as a systolic/diastolic blood pressure ≥130/80 mmHg or a self-report of taking antihypertensive drug. |
| Uncontrolled hypertension | Uncontrolled hypertension was defined as a systolic/diastolic blood pressure ≥130/80 mmHg among those who reported that they were taking antihypertensive drug. |
| Untreated hypertension | Untreated hypertension was defined as a systolic/diastolic blood pressure ≥130/80 mmHg among those who reported that they were not taking antihypertensive drug. |
| Chronic kidney disease | A glomerular filtration rate of <60 ml/min per 1.73 m^2^ or urinary albumin-creatinine ratio ≥30 mg/g. |
| Borderline and high cholesterol | If the cholesterol levels were 200–239 and ≥240mg/dl total cholesterol levels then it was defined as borderline and high cholesterol, respectively. Person reported taking antilipid drugs was also defined as having high total cholesterol. |
| Low high-density lipoprotein | A high-density lipoprotein lower than 40 mg/dL for men and lower than 50 mg/dL for women. Person reported taking antilipid drugs was also defined as having low high density lipoprotein |
| Diabetes | Diabetes was defined as the glycohemoglobin of ≥6.5%, if the fasting plasma glucose was ≥126 mg/dl or if the person was taking antidiabetic drugs. |
| Leisure time physical activity | None, low and high aerobic leisure time physical activity were defined as 0, >0 to <150, and ≥150 min/week, respectively. |
