# Supplementary material for: Association between height and hypertension among US adults: analyses of National Health and Nutrition Examination Survey 2007–18
Source: Clin Hypertens. 2021 Feb 26;27:6. doi: 10.1186/s40885-021-00164-4 (PMC7908753; doi:10.1186/s40885-021-00164-4)
Supplement: Supplementary file 2 — Additional file 2: Supplementary Table 2. Comparison of respondents according to height. [file 40885_2021_164_MOESM2_ESM.docx]

**Supplementary Table 2: Comparison of respondents according to height, NHANES 2007-18**

| **Variables** | **Q1** | **Q2** | **Q3** | **Q4** | **Overall** |
| --- | --- | --- | --- | --- | --- |
| **Age (in year)** |  |  |  |  |  |
| 20-39 | 29.9 (1,488) | 38.7 (1,881) | 43.1 (2,009) | 45.3 (2,319) | 39.9 (7,697) |
| 40-59 | 31.5 (1,611) | 35.1 (1,806) | 34.1 (1,747) | 36.4 (1,795) | 34.5 (6,959) |
| ≥60 | 38.5 (2,377) | 26.2 (1,773) | 22.9 (1,718) | 18.2 (1,411) | 25.6 (7,279) |
| **Gender** |  |  |  |  |  |
| Male | 4.4 (330) | 18.7 (1,458) | 56.6 (3,522) | 92.8 (5,189) | 47.1 (10,499) |
| Female | 95.6 (5,146) | 81.3 (4,002) | 43.4 (1,952) | 7.2 (336) | 52.9 (11,436) |
| **Race/Ethnicity** |  |  |  |  |  |
| Non-Hispanic White | 50.7 (1,578) | 62.4 (2,144) | 62.4 (2,237) | 72.0 (2,839) | 62.8 (8,798) |
| Non-Hispanic Black | 9.8 (767) | 12.8 (1,183) | 13.9 (1,328) | 12.3 (1,436) | 12.3 (4,714) |
| Mexican-American | 17.2 (1,439) | 10.4 (915) | 10.3 (831) | 5.7 (456) | 10.4 (3,641) |
| Other races/ethnicities | 22.3 (1,692) | 14.4 (1,218) | 13.4 (1,078) | 10.0 (794) | 14.5 (4,782) |
| **Family income to poverty ratio** |  |  |  |  |  |
| Low | 31.7 (2,159) | 27.1 (1,970) | 24.6 (1,763) | 21.0 (1,641) | 25.6 (7,533) |
| Middle | 35.7 (1,943) | 33.0 (1,865) | 33.4 (1,926) | 30.8 (1,881) | 33.0 (7,615) |
| High | 32.6 (1,374) | 39.9 (1,625) | 42.1 (1,785) | 48.2 (2,003) | 41.4 (6,787) |
| **Education level** |  |  |  |  |  |
| Below High School | 27.6 (2,051) | 19.0 (1,443) | 18.6 (1,432) | 14.7 (1,131) | 19.4 (6,057) |
| High School | 57.0 (2,725) | 60.9 (3,108) | 60.4 (3,069) | 61.7 (3,339) | 60.2 (12,241) |
| College Graduate or Above | 15.4 (700) | 20.1 (909) | 21.0 (973) | 23.6 (1,055) | 20.4 (3,637) |
| **Cholesterol level (in mg/dl)** |  |  |  |  |  |
| No high cholesterol | 38.3 (2,083) | 42.8 (2,271) | 45.5 (2,401) | 47.6 (2,546) | 44.0 (9,301) |
| Borderline elevated | 18.3 (992) | 18.9 (977) | 18.1 (942) | 16.2 (912) | 17.8 (3,823) |
| High cholesterol | 43.4 (2,401) | 38.2 (2,212) | 36.3 (2,131) | 36.1 (2,067) | 38.2 (8,811) |
| **High-density lipoprotein cholesterol (in mg/dl)** |  |  |  |  |  |
| Normal | 64.8 (3,443) | 66.3 (3,582) | 68.9 (3,750) | 67.2 (3,776) | 66.9 (14,551) |
| Low | 35.2 (2,033) | 33.7 (1,878) | 31.1 (1,724) | 32.8 (1,749) | 33.1 (7,384) |
| **Chronic kidney disease** |  |  |  |  |  |
| No | 78.0 (4,191) | 83.5 (4,428) | 85.9 (4,484) | 89.4 (4,706) | 84.7 (17,809) |
| Yes | 22.0 (1,285) | 16.5 (1,032) | 14.1 (990) | 10.6 (819) | 15.3 (4,126) |
| **Diabetes mellitus status** |  |  |  |  |  |
| No | 84.3 (4,416) | 87.5 (4,578) | 87.9 (4,599) | 87.9 (4,667) | 87.1 (18,260) |
| Yes | 15.7 (1,060) | 12.5 (882) | 12.1 (875) | 12.1 (858) | 12.9 (3,675) |
| **Smoker** |  |  |  |  |  |
| No | 84.2 (4,702) | 78.1 (4,315) | 73.7 (4,026) | 73.3 (3,902) | 76.9 (16,945) |
| Yes | 15.8 (774) | 21.9 (1,145) | 26.3 (1,448) | 26.7 (1,623) | 23.1 (4,990) |
| **Leisure time physical activity (in minutes)** |  |  |  |  |  |
| No/Low | 60.7 (3,513) | 52.0 (3,093) | 48.0 (2,896) | 45.7 (2,747) | 50.9 (12,249) |
| Some (<150 minutes) | 15.5 (803) | 16.6 (809) | 16.1 (809) | 14.5 (713) | 15.7 (3,134) |
| High (≥150 minutes) | 23.8 (1,160) | 31.4 (1,558) | 35.9 (1,769) | 39.7 (2,065) | 33.4 (6,552) |
| **Survey year** |  |  |  |  |  |
| 2007-10 | 31.0 (1,903) | 32.1 (1,964) | 31.7 (1,916) | 32.5 (2,008) | 31.9 (7,791) |
| 2011-14 | 33.2 (1,677) | 34.2 (1,801) | 34.5 (1,864) | 34.9 (1,894) | 34.3 (7,236) |
| 2015-18 | 35.8 (1,896) | 33.6 (1,695) | 33.8 (1,694) | 32.6 (1,623) | 33.8 (6,908) |

**NHANES:** National Health and Nutrition Examination Survey
