# Supplementary material for: Association between height and hypertension among US adults: analyses of National Health and Nutrition Examination Survey 2007–18
Source: Clin Hypertens. 2021 Feb 26;27:6. doi: 10.1186/s40885-021-00164-4 (PMC7908753; doi:10.1186/s40885-021-00164-4)
Supplement: Supplementary file 3 — Additional file 3: Supplementary Table 3. Comparison of respondents according to height among those who were taking blood pressure lowering drugs, NHANES 2007-18. [file 40885_2021_164_MOESM3_ESM.docx]

**Supplementary Table 3: Comparison of respondents according to height among those who were taking blood pressure lowering drugs, NHANES 2007-18**

| **Variables** | **Q1** | **Q2** | **Q3** | **Q4** | **Overall** |
| --- | --- | --- | --- | --- | --- |
| **Age (in year)** |  |  |  |  |  |
| 20-39 | 2.7 (45) | 4.4 (59) | 8.3 (88) | 9.4 (100) | 6.1 (292) |
| 40-59 | 25.6 (402) | 34.5 (450) | 35.2 (421) | 46.0 (489) | 35.1 (1,762) |
| 60+ | 71.7 (1,422) | 61.1 (997) | 56.5 (935) | 44.6 (746) | 58.8 (4,100) |
| **Gender** |  |  |  |  |  |
| Male | 3.1 (96) | 18.7 (411) | 61.2 (995) | 93.7 (1,266) | 43.0 (2,768) |
| Female | 96.9 (1,773) | 81.3 (1,095) | 38.8 (449) | 6.3 (69) | 57.0 (3,386) |
| **Race/Ethnicity** |  |  |  |  |  |
| Non-Hispanic White | 62.2 (684) | 66.2 (622) | 66.7 (625) | 76.0 (707) | 67.7 (2,638) |
| Non-Hispanic Black | 12.4 (354) | 19.1 (487) | 16.9 (432) | 14.2 (432) | 15.5 (1,705) |
| Mexican-American | 9.3 (355) | 5.2 (160) | 4.6 (138) | 3.2 (79) | 5.7 (732) |
| Other races/ethnicities | 16.1 (476) | 9.4 (237) | 11.9 (249) | 6.5 (117) | 11.1 (1,079) |
| **Family income to poverty ratio** |  |  |  |  |  |
| Low | 28.5 (709) | 24.2 (507) | 20.7 (434) | 18.1 (359) | 23.1 (2,009) |
| Middle | 40.0 (710) | 36.3 (556) | 32.7 (510) | 30.7 (460) | 35.1 (2,236) |
| High | 31.4 (450) | 39.5 (443) | 46.5 (500) | 51.2 (516) | 41.8 (1,909) |
| **Education level** |  |  |  |  |  |
| Below High School | 29.9 (761) | 22.5 (447) | 20.7 (426) | 16.3 (312) | 22.6 (1,946) |
| High School | 58.9 (933) | 63.6 (868) | 61.4 (800) | 63.6 (802) | 61.8 (3,403) |
| College Graduate/Above | 11.2 (175) | 13.8 (191) | 18.0 (218) | 20.0 (221) | 15.6 (805) |
| **Cholesterol level (in mg/dl)** |  |  |  |  |  |
| No high cholesterol | 19.8 (398) | 22.3 (353) | 23.3 (347) | 25.3 (324) | 22.6 (1,422) |
| Borderline elevated | 11.5 (210) | 12.2 (171) | 10.0 (126) | 8.8 (137) | 10.7 (644) |
| High cholesterol | 68.7 (1,261) | 65.5 (982) | 66.7 (971) | 65.9 (874) | 66.8 (4,088) |
| **High-density lipoprotein cholesterol (in mg/dl)** |  |  |  |  |  |
| Normal | 63.8 (1,159) | 61.9 (945) | 62.8 (924) | 59.8 (856) | 62.1 (3,884) |
| Low | 36.2 (710) | 38.1 (561) | 37.2 (520) | 40.2 (479) | 37.9 (2,270) |
| **Chronic kidney disease** |  |  |  |  |  |
| No | 59.2 (1,096) | 67.1 (966) | 66.3 (894) | 74.7 (904) | 66.7 (3,860) |
| Yes | 40.8 (773) | 32.9 (540) | 33.7 (550) | 25.3 (431) | 33.3 (2,294) |
| **Diabetes mellitus status** |  |  |  |  |  |
| No | 68.3 (1,210) | 69.0 (985) | 69.6 (922) | 65.8 (827) | 68.2 (3,944) |
| Yes | 31.7 (659) | 31.0 (521) | 30.4 (522) | 34.2 (508) | 31.8 (2,210) |
| **Smoker** |  |  |  |  |  |
| No | 87.5 (1,660) | 83.2 (1,252) | 78.5 (1,141) | 79.2 (1,028) | 82.3 (5,081) |
| Yes | 12.5 (209) | 16.8 (254) | 21.5 (303) | 20.8 (307) | 17.7 (1,073) |
| **Leisure time physical activity (in minutes)** |  |  |  |  |  |
| No/Low | 69.9 (1,321) | 64.6 (1,012) | 57.6 (880) | 59.9 (821) | 63.3 (4,034) |
| Some (<150 minutes) | 14.0 (262) | 14.9 (207) | 16.2 (219) | 13.8 (184) | 14.7 (872) |
| High (≥150 minutes) | 16.1 (286) | 20.6 (287) | 26.2 (345) | 26.3 (330) | 22.1 (1,248) |
| **Survey year** |  |  |  |  |  |
| 2007-10 | 29.8 (647) | 32.4 (541) | 30.8 (495) | 32.6 (496) | 31.4 (2,179) |
| 2011-14 | 34.2 (585) | 34.3 (490) | 36.0 (496) | 34.1 (418) | 34.6 (1,989) |
| 2015-18 | 36.0 (637) | 33.2 (475) | 33.2 (453) | 33.3 (421) | 34.0 (1,986) |

**NHANES:** National Health and Nutrition Examination Survey
