# Supplementary material for: Association between height and hypertension among US adults: analyses of National Health and Nutrition Examination Survey 2007–18
Source: Clin Hypertens. 2021 Feb 26;27:6. doi: 10.1186/s40885-021-00164-4 (PMC7908753; doi:10.1186/s40885-021-00164-4)
Supplement: Supplementary file 4 — Additional file 4: Supplemental Table 4. Interaction between height and age to investigate association of height with hypertension and untreated hypertension, NHANES 2007-18. [file 40885_2021_164_MOESM4_ESM.docx]

**Supplemental table 4: Interaction between height and age to investigate association of height with hypertension and untreated hypertension, NHANES 2007-18**

| **Variables** | **COR (95% CI)** | | **AOR (95% CI)** | |
| --- | --- | --- | --- | --- |
| **Height Category (in cm)** |  | |  | |
| Q1 (135.3-159.2) | Ref |  | Ref |  |
| Q2 (159.3-166.2) | 1.2* | (1.0,1.5) | 0.7 | (0.3,1.5) |
| Q3 (166.3-173.6) | 2.0*** | (1.6,2.4) | 1.3 | (0.6,2.8) |
| Q4 173.7-204.5) | 2.3*** | (1.8,2.9) | 1.5 | (0.7,3.0) |
| **Age (in year)** |  |  |  |  |
| 20-39 | Ref |  | Ref |  |
| 40-59 | 6.0*** | (4.8,7.6) | 1.1 | (0.6,2.2) |
| ≥60 | 19.4*** | (15.3,24.5) | 1.8 | (0.9,3.6) |
| **Interaction (Height Category * Age)** |  |  |  |  |
| Q2 * 20-39 | Ref |  | Ref |  |
| Q2 * 40-59 | 0.7* | (0.5,0.9) | 1.5 | (0.6,3.4) |
| Q2 * 60+ | 0.6*** | (0.4,0.8) | 1.2 | (0.5,2.7) |
| Q3 * 40-59 | 0.4*** | (0.3,0.6) | 0.9 | (0.3,2.1) |
| Q3 * 60+ | 0.3*** | (0.2,0.5) | 0.5 | (0.2,1.2) |
| Q4 * 40-59 | 0.4*** | (0.3,0.5) | 0.6 | (0.3,1.3) |
| Q4 * 60+ | 0.2*** | (0.1,0.3) | 0.3*** | (0.1,0.6) |
| **Gender** |  |  |  |  |
| Male | Ref |  | Ref |  |
| Female | 0.7*** | (0.6,0.8) | 0.7*** | (0.6,0.9) |
| **Race/Ethnicity** |  |  |  |  |
| Non-Hispanic White | Ref |  | Ref |  |
| Non-Hispanic Black | 1.9*** | (1.7,2.1) | 1.7*** | (1.5,2.1) |
| Mexican-American | 0.8*** | (0.7,0.9) | 1.2 | (0.9,1.5) |
| Other races/ethnicities | 0.9* | (0.8,1.0) | 1.2 | (1.0,1.4) |
| **Family income** |  |  |  |  |
| Low | Ref |  | Ref |  |
| Middle | 1 | (0.9,1.2) | 1 | (0.8,1.1) |
| High | 1.1 | (1.0,1.2) | 0.8* | (0.7,1.0) |
| **Education level** |  |  |  |  |
| Below High School | Ref |  | Ref |  |
| High School | 1 | (0.9,1.1) | 1 | (0.9,1.2) |
| College Graduate or Above | 0.8** | (0.6,0.9) | 0.8 | (0.6,1.0) |
| **Cholesterol level (in mg/dl)** |  |  |  |  |
| No high cholesterol (<200) | Ref |  | --- |  |
| Borderline elevated (200-239) | 1.3*** | (1.2,1.5) | --- |  |
| High cholesterol (≥240) | 1.7*** | (1.6,1.9) | --- |  |
| **High-density lipoprotein cholesterol (in mg/dl)** |  |  |  |  |
| Normal | Ref |  | --- |  |
| Low | 1.4*** | (1.2,1.5) | --- |  |
| **Chronic kidney disease** |  |  |  |  |
| No | Ref |  | --- |  |
| Yes | 2.2*** | (1.9,2.5) | --- |  |
| **Diabetes mellitus status** |  |  |  |  |
| No | Ref |  | Ref |  |
| Yes | 1.8*** | (1.6,2.1) | 1.3*** | (1.1,1.6) |
| **Smoker** |  |  |  |  |
| No | Ref |  | Ref |  |
| Yes | 0.9* | (0.8,1.0) | 0.8** | (0.6,0.9) |
| **Leisure time physical activity (in minutes)** |  |  |  |  |
| No | Ref |  | --- |  |
| Some (<150 minutes) | 0.9* | (0.7,1.0) | --- |  |
| ≥150 minutes | 0.8*** | (0.7,0.8) | --- |  |
| **Survey Period** |  |  |  |  |
| 2007-10 | Ref |  | Ref |  |
| 2011-14 | 1 | (0.9,1.1) | 0.9 | (0.7,1.0) |
| 2015-18 | 1.1 | (0.9,1.2) | 1.1 | (0.9,1.3) |

**p < 0.05, **p < 0.01, ***p < 0.001, AOR: Adjusted Odds Ratio, COR: Crude Odds Ratio,* ***NHANES****: National Health and Nutrition Examination Survey.*

*Adjusted for all variables in the column, variables significant in bivariate analyses were included in multivariable model*
